# The Ethics of Autonomous Clinical AI: Shifting From Model Accuracy to Model Impact

**DOI:** 10.2196/112246

**Published:** 2026-10-23

**Authors:** Clair Sullivan

**Affiliations:** 1 Queensland Digital Health Centre (QDHeC), University of Queensland, Level 5, UQ Health Sciences Building, Brisbane, Queensland, 4006, Australia, 61 0400669296

**Keywords:** autonomous artificial intelligence, autonomous AI, clinical trials, research ethics, health equity, implementation

## Abstract

The ethics of autonomous clinical AI are controversial. Traditional ethics assessments have focused on AI model performance rather than health system outcomes. Nichol and colleagues translate established research ethics principles into 9 practical recommendations for trials of autonomous clinical AI. Their findings support an evolution in clinical trial design. The accuracy and performance of the model is important, but the ethical unit of evaluation should be the complete care pathway, with prespecified obligations for subgroup performance, follow-up care, system failures, and model change.

## A Timely Shift Beyond Accuracy

Clinical trials of AI have often treated the algorithm as the intervention and diagnostic performance as the principal outcome. Nichol and colleagues [1] challenge that narrow frame. Their modified Delphi study applied the 7 ethical principles described by Emanuel et al [2] to autonomous AI trials and produced 9 recommendations spanning data transparency, bias, generalizability, stakeholder engagement, consent, standards of care, downstream access, and cost. The central message is important. An accurate autonomous AI model has little social or clinical value if it does not improve health system outcomes.

Panelists did not agree that autonomous AI creates wholly new ethical problems. Bias, inadequate access, weak consent, and poorly chosen comparators predate AI. Yet autonomy can amplify these familiar risks. A clinician may no longer review each result, errors can be repeated at scale, performance can change across sites, and a positive screen can transfer clinical and financial burdens to patients or services that lack capacity.

## The Care Pathway Is the Ethical Unit of Evaluation

The paper’s most important contribution is its emphasis on health system outcomes rather than model accuracy. In the diabetic retinopathy example, a higher screening rate is an incomplete measure of benefit. A trial should also determine whether people with positive results receive confirmatory assessment, enter treatment when indicated, and avoid preventable loss to follow-up. It should measure time to care, patient burden, false-positive investigations, and the distribution of each outcome across relevant groups. Otherwise, an apparently successful intervention may improve detection while widening the gap between diagnosis and treatment.

This pathway-based approach also sharpens comparator selection. The relevant comparison is not simply AI accuracy versus clinician accuracy. It is the AI-enabled pathway versus the incumbent care pathway. These care pathways include patient reach, waiting time, referral completion, safety-netting, and opportunity costs. An autonomous system that performs only one task well, such as identifying retinopathy, may not identify additional findings that traditional screening is able to report, such as a doctor detecting an incidental finding of papilledema at a routine retinal screening. Trial protocols should therefore specify which functions are replaced, retained, or added and which patient-relevant outcome supports the claim of clinical value.

## Consensus Must Become Prespecified Obligations

The 9 recommendations are strong, but terms such as bias assessment, performance, and equity assessment still leave decisive choices to investigators. Ethical guidance becomes enforceable only when protocols define thresholds and actions in advance. A subgroup performance difference should trigger a stated response, such as model modification, restricted use, enhanced monitoring, or nondeployment. Sites should document referral capacity before enrollment, and trial budgets should identify who pays for confirmatory care and treatment. Independent reviewers should be able to determine whether these conditions were met rather than infer them after favorable results are known.

Existing AI reporting guidance offers a foundation. SPIRIT-AI (Standard Protocol Items: Recommendations for Interventional Trials–Artificial Intelligence) and CONSORT-AI (Standard Protocol Items: Recommendations for Interventional Trials–Artificial Intelligence) extend protocol and trial-reporting standards for interventions involving AI, while DECIDE-AI (Developmental and Exploratory Clinical Investigations of Decision Support Systems Driven by Artificial Intelligence) addresses early live clinical evaluation [3-5]. The Delphi recommendations add a needed ethical layer, but the next step is integration. Protocol registries, ethics applications, and statistical analysis plans should contain a common set of pathway outcomes, equity analyses, escalation rules, and accountability assignments. This can be challenging for those AI developers who lack health system connections and expertise.

## Autonomy Belongs to a Changing Clinical System

Autonomy should also be treated as a property of the deployed system, not a permanent label attached to a model. Human involvement may remain in image acquisition, communication of results, referral, exception handling, and surveillance. Trials need to describe these roles, identify who can override or suspend the system, and test failure modes such as unusable inputs, uncertain outputs, workflow interruptions, and delayed follow-up. The ethical question is not only whether a model performs well, but whether responsibility remains visible when no specialist reviews the output at the point of care.

Versioning adds another difficulty. A trial evaluates a specific model in a specific workflow, whereas deployment may include software updates, changes in devices, and shifts in patient populations. Good machine learning practice and regulatory approaches to planned model changes recognize this life cycle problem [6,7]. Ethical review should therefore specify which changes require revalidation, renewed consent information, additional subgroup analysis, or independent review.

## From Recommendations to a Trial Standard

The Delphi panel was small, based entirely in the United States, and anchored in diabetic retinopathy screening. The recommendations may not transfer unchanged to therapeutic, triage, or management tasks or to health systems with different financing and referral structures. Broader work should include patients who have experienced AI-mediated care, communities likely to bear implementation risks, frontline staff, payers, and regulators from varied settings.

The next task is to convert these 9 recommendations into a prospective, auditable standard for trial design. Autonomous AI should be judged by the pathway it creates, the care patients can actually reach, and the safeguards that remain when the model or setting changes. Accuracy is necessary evidence. It is not the end point of ethical evaluation.

## Abbreviations

CONSORT-AI: Consolidated Standards of Reporting Trials for Artificial Intelligence
DECIDE-AI: Developmental and Exploratory Clinical Investigations of Decision Support Systems Driven by Artificial Intelligence
SPIRIT-AI: Standard Protocol Items: Recommendations for Interventional Trials–Artificial Intelligence
